# Pre-exercise Carbohydrate Drink Adding Protein Improves Post-exercise Fatigue Recovery

**DOI:** 10.3389/fphys.2021.765473

**Published:** 2021-11-22

**Authors:** Albert Yi-Wey Tan, Sareena-Hanim Hamzah, Chih-Yang Huang, Chia-Hua Kuo

**Affiliations:** ^1^Centre for Sport and Exercise Sciences, University of Malaya, Kuala Lumpur, Malaysia; ^2^Cardiovascular and Mitochondrial Related Disease Research Center, Hualien Tzu Chi Hospital, Buddhist Tzu Chi Since Medical Foundation, Hualien, Taiwan; ^3^Graduate Institute of Biomedical Sciences, China Medical University, Taichung, Taiwan; ^4^Department of Medical Research, China Medical University Hospital, China Medical University, Taichung, Taiwan; ^5^Department of Biotechnology, Asia University, Taichung, Taiwan; ^6^Center of General Education, Buddhist Tzu Chi Medical Foundation, Tzu Chi University of Science and Technology, Hualien, Taiwan; ^7^Laboratory of Exercise Biochemistry, University of Taipei, Taipei, Taiwan

**Keywords:** free radical scavenging capacity, plant-based protein, reduced-to-oxidized glutathione, GSH, GSSG, soy, ferric reducing antioxidant power, FRAP

## Abstract

**Purpose:** This study aimed to assess the requirement of protein in pre-exercise carbohydrate drinks for optimal endurance performance at high intensity and post-exercise fatigue recovery.

**Methods:** Endurance performance at 85% $\dot{\text{V}}\,{\text{O}}_{2}$_peak_ of young men (age 20 ± 0.9 years, $\dot{\text{V}}\,$_2peak_ 49.3 ± 0.3 L/min) was measured for two consecutive days using cycling time to exhaustion and total work exerted 2 h after three isocaloric supplementations: RICE (50 g, protein: 1.8 g), *n* = 7; SOY + RICE (50 g, protein: 4.8 g), *n* = 7; and WHEY + RICE (50 g, protein: 9.2 g), *n* = 7.

**Results:** Endurance performance was similar for the three supplemented conditions. Nevertheless, maximal cycling time and total exerted work from Day 1 to Day 2 were improved in the WHEY + RICE (+21%, *p* = 0.05) and SOY-RICE (+16%, *p* = 0.10) supplemented conditions, not the RICE supplemented condition. Increases in plasma interleukin-6 (IL-6) were observed 1 h after exercise regardless of supplemented conditions. Plasma creatine kinase remained unchanged after exercise for all three supplemented conditions. Increases in ferric reducing antioxidant power (FRAP) after exercise were small and similar for the three supplemented conditions.

**Conclusion:** Adding protein into carbohydrate drinks provides no immediate benefit in endurance performance and antioxidant capacity yet enhances fatigue recovery for the next day. Soy-containing carbohydrate drink, despite 50% less protein content, shows similar fatigue recovery efficacy to the whey protein-containing carbohydrate drink. These results suggest the importance of dietary nitrogen sources in fatigue recovery after exercise.

## Introduction

Protein is not the primary energy substrate supporting the high-intensity exercise. However, protein supplementation is known to accelerate healing during inflammation (Thomas, 1997). Inflammation is the innate immune mechanism responsible for recovering skeletal muscle from damage (Tidball, 2017). During high-intensity exercise, muscle damage is inevitably occurring which immediately triggers cell regeneration in contracting muscle (Wu et al., 2019a; Lee et al., 2021). Nitrogen from protein and amino acids is essential for DNA synthesis and cell regeneration during recovery after intensive exercise (Yang et al., 2018; Tryfidou et al., 2020).

Carbohydrate is considered the most important energy source for ATP synthesis contributing to prolonged high-intensity endurance exercise (Coyle et al., 1986; Hawley and Leckey, 2015). Post-exercise carbohydrate supplementation with a small amount of protein has been shown to accelerate recovery in endurance performance 4 h following cycling relative to carbohydrate supplementation without protein (Saunders, 2007; Hall et al., 2013). Low-protein supplementation delays the resolution of inflammation after muscle-damaging exercise (Yang et al., 2018). However, the benefit of protein addition on endurance performance is abolished when antioxidants are included in the supplement (Romano-Ely et al., 2006), suggesting that free radicals originated from inflammation (phagocytosis) mediate the fatigue recovery. Amino acid is known to activate phagocytosis with increased free radical production *in vitro* (Zhenyukh et al., 2017). Free radicals are found essential for training adaptation against aerobic exercise (Gomez-Cabrera et al., 2008). Soy is a plant-based protein source containing antioxidants (Box et al., 2005). Nevertheless, soy supplementation does not seem to affect pro-inflammatory interleukin-6 (IL-6) levels in randomized clinical trials (Beavers et al., 2009). It remains unknown whether adding natural soy into pre-exercise carbohydrate drinks can influence free radicals, endurance performance, and post-exercise fatigue recovery.

This study aimed to address the question of whether we should include protein sources (whey protein or natural soy) into pre-exercise carbohydrate drinks to optimize endurance performance and post-exercise fatigue recovery. We also examined the association between biomarkers of circulating inflammation/free radicals and endurance performance during fatigue recovery. Timing of pre-exercise supplementation seems to be important for endurance performance. For example, cycling and running times to exhaustion at moderate-to-high intensity [70% maximum oxygen consumption ($\dot{\text{V}}\,$_2max_)] improve when carbohydrate was supplemented 2–3 h before the exercise challenge (Schabort et al., 1999; Chryssanthopoulos et al., 2002; Chen et al., 2009). However, studies examining endurance performance following carbohydrate supplementation within 1 h before continuous (Hargreaves et al., 1987; Febbraio et al., 2000) and intermittent exercise (Pritchett et al., 2008) show mixed results. In this study, a protein-containing carbohydrate beverage was orally given 2 h before high-intensity exercise at 85% $\dot{\text{V}}\,$_2max_. We hypothesized (1) enhanced endurance performance after consumption of a protein-containing (whey protein or soy) carbohydrate beverage compared with an isocaloric carbohydrate alone drink, (2) attenuated performance enhancement after pre-exercise soy-containing carbohydrate beverage associated with higher free radical scavenging capacity, and (3) improved post-exercise fatigue recovery, assessed by the same endurance performance test on Day 2 after consumption of a protein-containing carbohydrate beverage (whey protein or soy) compared with an isocaloric carbohydrate alone drink.

## Materials and Methods

### Participants

Seven physically active men (age 20.0 ± 0.9 years; height 167.7 ± 4.4 cm; body mass 56.4 ± 4.8 kg; and $\dot{\text{V}}\,$_2peak_ 49.3 ± 0.3 L/min) with exercise habit > three times per week were recruited for this study. The participants signed informed consent after a verbal and written briefing on the procedures of this study including possible risks and discomforts involved. Then, they were completed a Physical Activity Readiness Questionnaire (PAR-Q) form prior to this study. Exclusion criteria are vegetarian, smokers, on a weight-reducing diet, consuming medication, or drugs, diagnosed with neurological, metabolic, and/or cardiovascular diseases, having an injury, and presenting high risk for performing maximal intensity exercises. University of Malaya Research Ethics Committee approved this study. The sample size was calculated using G-Power version 3.1.9.2 (Informer Technologies, Inc. United States) on a study by Romano-Ely et al. (2006). The G-Power indicated that a minimum sample of seven produced 95% CI with an effect size of *f* = 1.10, α = 0.05, and β = 0.80.

### Drink

Three isocaloric beverages (500 ml) were used in this study. The nutritional content of the drinks is shown in Table 1. RICE only beverage consists of 6% rice (30 g) and 4% cane sugar (20 g). SOY + RICE beverage contained 2% soybean (10 g), 4% rice (20 g), and 4% cane sugar (20 g). WHEY + RICE beverage contained 2% whey protein (10 g), 4% rice (20 g), and 4% cane sugar (20 g).

**TABLE 1:** Nutritional content of pre-exercise drinks.

Macronutrients	RICE (50 g)	SOY + RICE (50 g)	WHEY + RICE (50 g)
**Weight (g)**			
Carbohydrate	44.3	38.5	36.8
Protein	1.8	4.8	9.2
Fat	0.2	1.8	0.8
Total	46.3	45.1	46.8
**Calories (kcal)**			
Carbohydrate	185.9	161.6	154.4
Protein	7.9	20.7	39.6
Fat	2.0	16.7	7.5
Total	195.8	199.0	201.5

### Study Design

**FIGURE 1:**
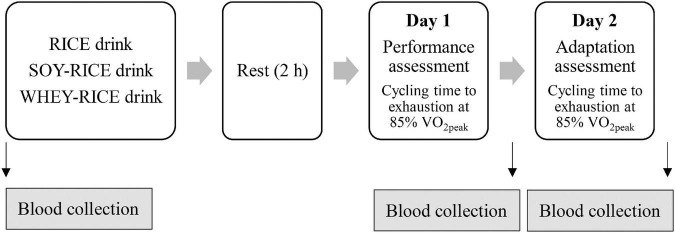
The experimental design consisted of three isocaloric carbohydrate drinks (Table 1) supplemented 2 h before endurance performance test at 85% $\dot{\text{V}}\,$_2peak_: RICE alone drink (*n* = 7), SOY-RICE drink (*n* = 7), and WHEY-RICE drink (*n* = 7) with a 7-day washout period between trials. The magnitude of fatigue recovery is indicated by performance improvement from Day 1 to Day 2 against the same cycling performance test. Arrow indicates the time of blood collection for measurements of creatine kinase (CK), interleukin-6 (IL-6), reduced-to-oxidized glutathione (GSH-to-GSSG ratio), and ferric reducing antioxidant power (FRAP). RICE alone drink (carbohydrate 95%, protein 4%, and fat 1%), SOY-RICE drink (carbohydrate 82%, protein 10%, and fat 8%), and WHEY-RICE drink (carbohydrate 76%, protein 20%, and fat 4%) in calorie.

The experimental design to assess the ergogenic effect of pre-exercise supplements (195–200 kcal) on high-intensity endurance performance (cycling time to exhaustion at 85% $\dot{\text{V}}\,$_2peak_ and total work exerted) and recovery against the same exercise challenge is shown in Figure 1. Participants were randomized into one of the three beverage supplemented conditions in a counterbalanced order. The participants were fasted for 12 h before consuming 500 ml (1) rice mixed with soybean (SOY + RICE), (2) rice mixed with whey protein (WHEY + RICE), or (3) rice alone (RICE) as the control condition 2 h before the endurance performance test on a cycle ergometer (Day 1). Participants repeated the same experimental protocol with the same beverage on the next day (Day 2) to determine recovery efficacy. The three supplemented conditions were separated by a 1-week washout period. They were informed to refrain from taking any soy-based or whey protein-related supplements 2 days before the first trial and between Day 1 and Day 2 until the completion of all supplemented conditions. The participants were asked to limit themselves to activities of daily living and slow walking or cycling for personal transport 2 days before the trials and between Day 1 and Day 2 for each trial. All cycling trials with different supplements were performed under consistent experimental procedures under the same environmental conditions. A physician was on duty to monitor the safety of challenged participants during all the trials.

The participants were required to visit the laboratory on eight occasions. Each visit comprised of familiarization, sub-maximum cycling, and $\dot{\text{V}}\,$_2max_ test, and three back-to-back cycling to exhaustion at 85% $\dot{\text{V}}\,$_2max_ sessions with a 1-week period gap between them in a counter-balanced order. For the $\dot{\text{V}}\,$_2max_ test, after a 30-min rest from the sub-maximum cycling test, the participant pedaled at 75 W for 1 min with 25 W increment every 1 min until volatile exhaustion. The $\dot{\text{V}}\,$_2max_ was determined when the participant met at least two out of the following three criteria: (1) respiratory exchange ratio (RER) above 1.1, (2) VO_2_ reached a plateau, and (3) 95% predicted maximum heart rate (HR_max_). The maximum value of oxygen consumption was recorded as $\dot{\text{V}}\,$_2peak_ if no plateau is observed. The aerobic power at 85% $\dot{\text{V}}\,$_2peak_ of each participant was obtained using a regression formula between pedaling power and % $\dot{\text{V}}\,$_2peak_.

### Experimental Protocol

Participants arrived at the laboratory after a 12-h overnight fast, at approximately 8–9 h (Day 1). They were weighed using bioelectrical impedance analysis (InBody, United States) and a cannula (G-15, Venflon) was inserted in an antecubital vein by a phlebotomist. After a 10-min rest and blood collection, participants consumed one of the isocaloric RICE, SOY + RICE, or WHEY + RICE beverages. The participants were asked to stay within the testing area and remained sedentary (i.e., sitting, reading, and studying) for 2 h before a brief warm-up for 5 min. Then, participants were cycled on a Monark 839 E ergometer (Vansbro, Sweden) at a workload equivalent to 85% $\dot{\text{V}}\,$_2peak_ until volitional exhaustion. Heart rate was monitored throughout the test using Polar FT4M (Polar Electro, Finland). Exhaustion is defined as the point at which participants can no longer maintain the cycling load. At this point, the rate of perceived exertion (RPE) was recorded using the Borg scale (Borg, 1982). The workload was recorded in kilopond per minute, and the total duration spent was used to calculate the total work done (kilopond). Blood samples were taken immediately (0 min) and 1 h post-exercise. To assess the magnitude of recovery against the exercise challenge from Day 1, the same protocol was repeated 24 h later (Day 2).

### Free Radical Scavenging Capacity

Venous blood samples were collected into precooled appropriate tubes (EDTA, Heparin or plain) and centrifuged at 3,000 rpm for 15 min at 4°C before being assayed for ferric reducing antioxidant power (FRAP) (OxiSelect™, Inc., United States), glutathione (GSH and GSSG) (BioVision, United States), IL-6 (eBioscience, Vienna, Austria) with ELISA readers (Tecan Genios, Salzburg, Austria) while plasma creatine kinase (CK) was analyzed enzymatically using a benchtop DT-60IIanalyzer (Johnson and Johnson, NY, United States).

### Statistical Analyses

All values are expressed as a difference from baseline (mean ± SE). A one-way ANOVA with repeated measures was used for comparisons between three time points for endurance and plasma variables. A paired *t*-test with Bonferroni’s correction was used to compare treatment differences between Day 1 and Day 2. The probability of a type I error less than 5% is considered statistically significant and 5–10% is considered moderately significant. Cohen’s *d* was used to indicate the effect size of intervention on recovery (endurance performance improvement from Day 1 to Day 2).

## Results

**FIGURE 2:**
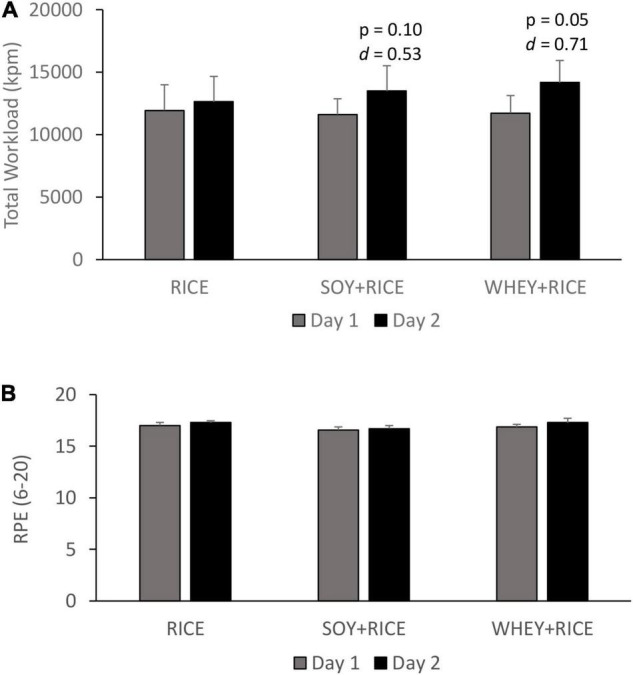
Protein addition into carbohydrate drinks provides no immediate benefit in high-intensity endurance performance (85% $\dot{\text{V}}\,{\text{O}}_{2}$_peak_) than carbohydrate alone drink (*n* = 7). Total work exerted **(A)** was not immediately improved by protein addition, but recovery (performance on Day 2) was elevated only for the protein added condition. The rate of perceived exertion (RPE) at exhaustion was similar for the three supplemented trials **(B)**. RICE alone drink (carbohydrate 95%, protein 4%, and fat 1%), SOY-RICE drink (carbohydrate 82%, protein 10%, and fat 8%), and WHEY-RICE drink (carbohydrate 76%, protein 20%, and fat 4%) in calorie.

**FIGURE 3:**
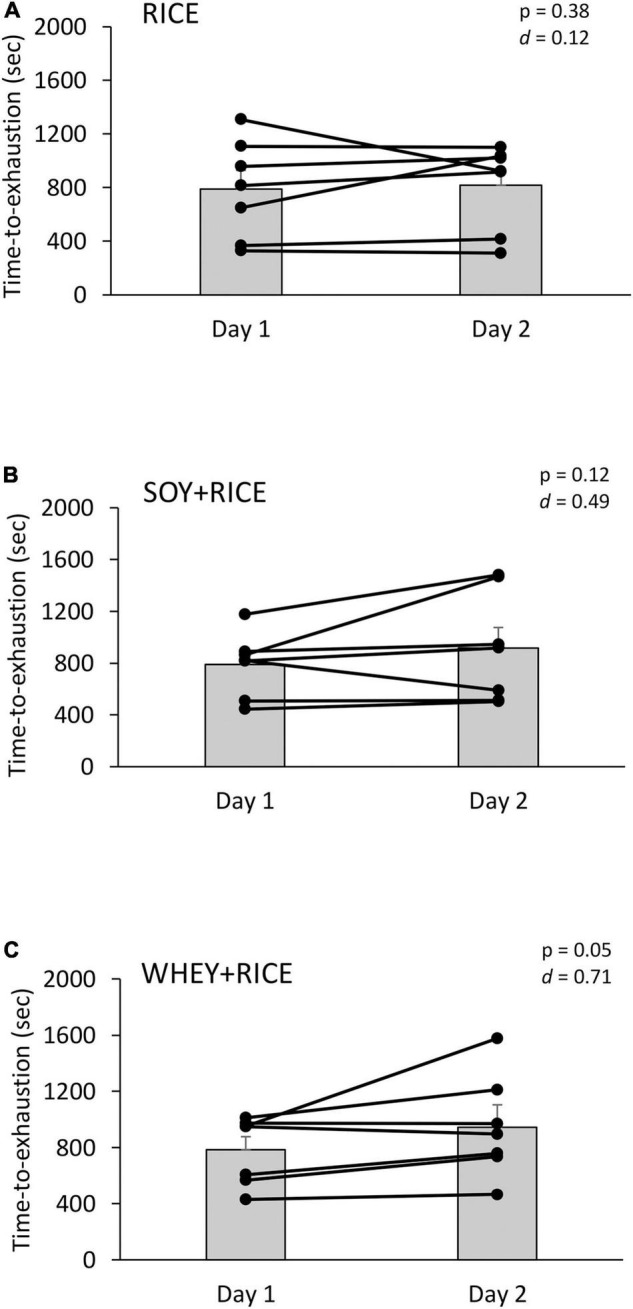
Individual performance improvements from Day 1 to Day 2 against the same exhaustive cycling test at 85% $\dot{\text{V}}\,{\text{O}}_{2}$_peak_ for RICE alone drink (*n* = 7) **(A)**, SOY-RICE drink (*n* = 7) **(B)**, and WHEY-RICE drink (*n* = 7) **(C)**. RICE alone drink (carbohydrate, 95%, protein, 4%; fat, 1%), SOY-RICE drink (carbohydrate, 82%, protein, 10%; fat, 8%), and WHEY-RICE drink (carbohydrate, 76%, protein, 20%; fat, 4%) in calorie.

On Day 1, high-intensity endurance performance (Figure 2) indicated by time to exhaustion (Figure 3) 2 h following the pre-exercise beverage ingestion was similar for the RICE, SOY + RICE, and WHEY + RICE supplemented conditions. The total work exerted (in kilopond) on Day 1 (Figure 2A) and RPE at exhaustion were similar among the three supplemented conditions (Figure 2B).

A significant difference between Day 1 and Day 2 indicates the magnitude of recovery from the Day 1 exercise. On Day 2, the total exerted cycling work increased by 5.7, 16.2, and 20.7% above Day 1 for the RICE, SOY-RICE, and WHEY-RICE supplemented conditions, respectively. Significant improvements in cycling time to exhaustion and total work exerted (in kilopond) from Day 1 were observed during the WHEY + RICE trial and to a moderate extent during the SOY + RICE trial. No improvement in cycling time to exhaustion and total workload between Day 1 and Day 2 was observed in the RICE trial. The protein content of SOY + RICE was only half of the WHEY + RICE drink (Table 1). The magnitude of improvements was similar for the SOY + RICE and WHEY + RICE supplemented conditions.

**FIGURE 4:**
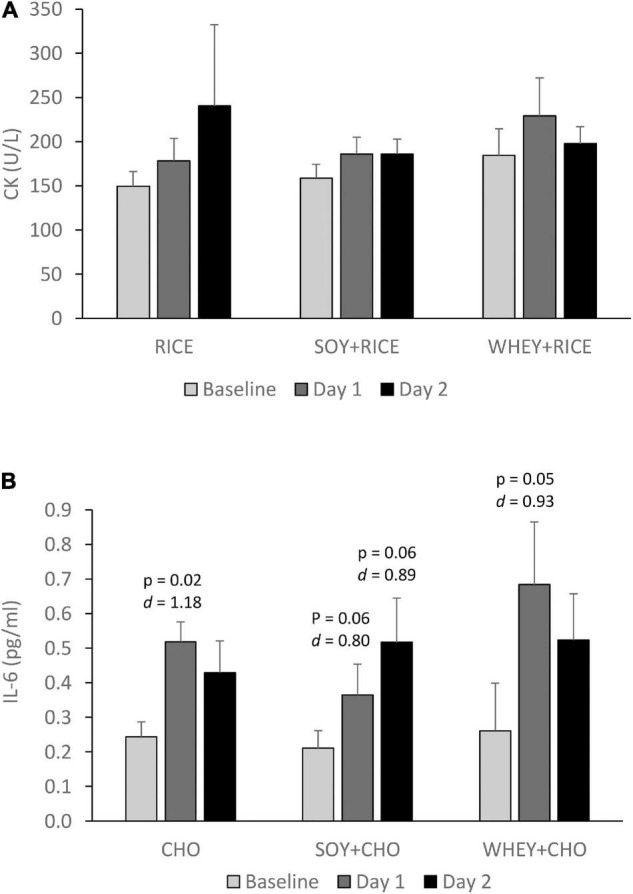
Plasma IL-6 **(A)** and CK **(B)** responses 1 h after exercise are similar for RICE alone drink (*n* = 7), SOY-RICE drink (*n* = 7), and WHEY-RICE drink (*n* = 7) at Day 1 and Day 2. RICE alone drink (carbohydrate, 95%, protein, 4%; fat, 1%), SOY-RICE drink (carbohydrate, 82%, protein, 10%; fat, 8%), and WHEY-RICE drink (carbohydrate, 76%, protein, 20%; fat, 4%) in calorie.

Exercise-induced muscle damage and pro-inflammatory response, mirrored by plasma CK and IL-6 levels, were measured 1 h following exercise on Day 1 and Day 2 under the RICE, SOY + RICE, and WHEY + RICE supplemented conditions (Figure 4). No significant time effect was detected in plasma CK after the exhausted bout of cycling for the three supplemented conditions (Figure 4A). IL-6 was consistently elevated following exercise for the three supplemented conditions (Figure 4B). Differences in post-exercise IL-6 were not significant on Day 1 and Day 2 for all conditions.

**FIGURE 5:**
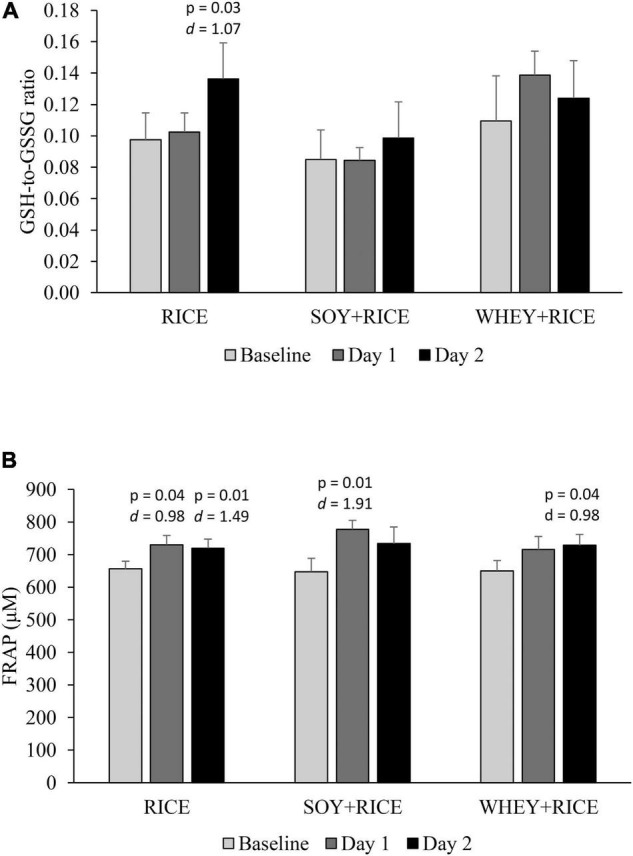
Post-exercise antioxidant capacity (*n* = 7). Plasma reduced-to-oxidized glutathione (GSH-to-GSSG ratio) **(A)** and FRAP **(B)**, measured 1 h post-exercise, are similar for the three supplemented trials on the baseline, Day 1 and Day 2. Marginal time effect was found for FRAP but not for GSH-to-GSSG ratio **(B)**. RICE alone drink (carbohydrate 95%, protein 4%, and fat 1%), SOY-RICE drink (carbohydrate 82%, protein 10%, and fat 8%), and WHEY-RICE drink (carbohydrate 76%, protein 20%, and fat 4%) in calorie.

Post-exercise free radical scavenging capacity was indicated by the GSH-to-GSSG ratio and FRAP in plasma, measured 1 h post-exercise (Figure 5). The GSH-to-GSSG ratio on Day 2 was increased above baseline for the RICE supplemented condition, but not significant for the SOY + RICE and WHEY + RICE supplemented conditions (Figure 5A). Post-exercise plasma FRAP under the RICE, SOY + RICE, and WHEY + RICE supplemented conditions are presented in Figure 5B. A trend of a small increase in plasma FRAP was observed 1 h after exercise regardless of supplemented conditions.

## Discussion

Pre-exercise carbohydrate supplementation (2–3 h prior to exercise) improves a time-to-exhaustion running performance (Chryssanthopoulos et al., 2002). This study asked the question of whether adding a small amount of protein provides immediate benefit for endurance performance and subsequent fatigue recovery on Day 2 against the same exercise challenge, compared with isocaloric carbohydrate alone drink. In this study, no immediate performance enhancement effect of protein addition was observed. However, endurance performance against the same cycling test was significantly improved on Day 2 when protein was included in the pre-exercise carbohydrate drink, without a significant difference between whey protein isolate (20% improvement) and natural soy (16% improvement). Since protein is not the major fuel to sustain high-intensity endurance exercise, the finding of this study implicates a requirement of dietary nitrogen for post-exercise recovery.

The result of this study demonstrates a carry-over effect of pre-exercise protein supplementation for high-intensity endurance performance on the next day. The underlying mechanism for this delayed effect remains unclear. Exercise challenge causes tissue damage and triggers cell regeneration after mobilization of hematopoietic progenitor cells and endothelial progenitor cells into the sites of damage during inflammation (Krüger et al., 2015; Wu et al., 2019a,b; Lee et al., 2021). The cell regeneration process requires nitrogen source and time for DNA synthesis (Hosios et al., 2016), which might explain no immediate ergogenic effect of protein addition into the protein-containing carbohydrate drink, but a far-reaching improvement in high-intensity endurance performance on Day 2. We speculated that supplementing protein short after exercise can also produce a similar benefit. In a short-term training study, significant improvements in running performance with a less subjective feeling of performance capacity loss were observed when protein-containing carbohydrate supplements were orally given after exercise, compared with carbohydrate alone supplements (Hansen et al., 2015). It is generally known that amino acids and proteins exert a psychological effect on the brain under stressed conditions. Milk protein significantly improves mood and cortisol levels after stress among normal participants (Markus et al., 2000). Therefore, we could not preclude the possibility that the observed delayed effect on fatigue recovery is mediated by its dual effects on the brain and muscle.

Interleukin-6 is a pro-inflammatory cytokine produced as part of a signal that triggers healing after challenge. Exercise-induced recovery in endurance performance requires IL-6 (McGinnis et al., 2015; Marasco et al., 2018) and free radical production (Gomez-Cabrera et al., 2008) during inflammation. The inflammation process, involved with the elimination of injured tissue by phagocytosis followed by a protracted cell regeneration, is essential for recovery against physical challenge (Tidball, 2017). Increased leukocyte infiltration, satellite cell replenishment, and increased free radicals are normally observed in challenged muscle after aerobic cycling exercise without observable changes in plasma CK and GSH-to-GSSG ratio (Wu et al., 2019a,b). However, in this study, we do not find the difference in responses of IL-6, GSH-to-GSSG ratio, and FRAP against exercise among the three pre-exercise supplemented conditions, albeit better exercise recovery outcomes after consumption of protein-added rice supplements. This suggests that nutritional nitrogen source is a limiting factor for post-exercise recovery.

Soy supplementation has been shown to lower systemic inflammation (Mangano et al., 2013) and oxidative stress (Tikkanen et al., 1998; Jenkins et al., 2000) for non-exercise individuals. However, in this study, we could not observe the suppressive effect of soy addition on exercise-induced IL-6 elevation as well as the difference in antioxidant markers. The absence of decreased plasma GSH-to-GSSG ratio together with slightly elevated FRAP suggests that the oxidative stress produced during the exercise test is sufficiently accommodated by endogenous antioxidant capacity in these young men. In addition, the previously observed antioxidant effect of soy may not be mediated by the neutralization of free radicals.

It is worthy to note that the SOY-RICE drink used in this study has only 50% of protein of the WHEY-RICE drink. However, the performance improvement on Day 2 was similar for the SOY-RICE and WHEY-RICE drinks. We could not preclude the possibility that other ingredients in natural soy than protein play a role in facilitating the physiological recovery against the exercise challenge.

A major limitation of this study is the low sample size. Furthermore, the potential influence of different fat contents is another inevitable limitation for such type of dietary study. Therefore, the generalization of the knowledge produced from the current work should be interpreted with caution.

## Conclusion

The result of this study demonstrated that consuming carbohydrate drinks (50 g) containing either natural soy or whey protein isolate 2 h before exercise provides no immediate benefit in performance enhancement at high intensity compared with isocaloric carbohydrate alone drink. However, fatigue recovery in endurance performance can be improved on Day 2 by including dietary protein into the pre-exercise carbohydrate drink. This study also provides encouraging evidence that natural protein-enriched soy supplementation, with less soy protein (10.6%), could produce a similar benefit in fatigue recovery as whey protein (19.7%).

## Data Availability Statement

The raw data supporting the conclusions of this article will be made available by the authors, without undue reservation.

## Ethics Statement

The studies involving human participants were reviewed and approved by the University of Malaya Research Ethics Committee (UM.TNC2/RC/H&E/UMREC-115), and all participants gave written consent.

## Author Contributions

AT and C-YH, and C-HK designed the experiments. AT performed the experiments and statistical analyses. AT, S-HH, C-YH, and C-HK wrote the manuscript. All authors contributed to the article and approved the submitted version.

## Conflict of Interest

The authors declare that the research was conducted in the absence of any commercial or financial relationships that could be construed as a potential conflict of interest. The handling editor declared a past co-authorship with one of the author C-HK.

## Publisher’s Note

All claims expressed in this article are solely those of the authors and do not necessarily represent those of their affiliated organizations, or those of the publisher, the editors and the reviewers. Any product that may be evaluated in this article, or claim that may be made by its manufacturer, is not guaranteed or endorsed by the publisher.
